# Supplementary material for: Atomic level fluxional behavior and activity of CeO2-supported Pt catalysts for CO oxidation
Source: Nat Commun. 2021 Oct 4;12:5789. doi: 10.1038/s41467-021-26047-8 (PMC8490411; doi:10.1038/s41467-021-26047-8)
Supplement: Supplementary file 1 — Supplementary Information [file 41467_2021_26047_MOESM1_ESM.pdf]

Atomic Level Fluxional Behavior and Activity of CeO_2-supported Pt Catalysts for CO Oxidation

Joshua L. Vincent[1], and Peter A. Crozier[1]*

[1]*School for Engineering of Matter, Transport, and Energy, Arizona State University, Tempe, Arizona 85281*

*Corresponding Author:
Peter A. Crozier
Engineering G Wing, #301
501 E. Tyler Mall
Tempe, AZ 85287-6106
Tel: 480 965 2934
Fax: 480 727 9321
Email: crozier@asu.edu

Supplementary Note 1: Catalyst preparation and characterization

The catalyst consists of Pt nanoparticles supported on CeO_2 nanoparticles having a generally cubic morphology. Nanostructured CeO_2 cubes were chosen as model support due to their shape which facilitates the identification of on-axis particles during high resolution imaging. Methods describing the synthesis procedures for various CeO_2 morphologies have been published previously[1]. Generally, the synthesis involves the precipitation of crystalline CeO_2 from an aqueous basic solution when heated autogenously in a hydrothermal vessel. In a typical synthesis, 1.38 g of $Ce(NO_3)_3{\bullet}6H_2O$ dissolved in 8 mL of deionized (DI) water was added to a 60 mL solution of 12 M NaOH, which was stirred for 30 minutes. The resultant pale-white slurry was added to an 85 mL Teflon-lined autoclave and heated for 24 hours at 200 °C. Upon cooling to room temperature, the fluffy white precipitates were isolated by filtration, washed multiple times with DI water, and dried at 60 °C in air overnight. After drying, the powders were calcined in air at 350 °C for 4 hours.

An impregnation technique was used to deposit 17 wt.% Pt onto the CeO_2 nanocubes. A high weight loading of metal was desired to reduce the amount of time spent searching for Pt nanoparticles close to a zone-axis orientation during *in situ* and *operando* TEM experiments. In brief, a volume of CeO_2 powder was weighed, then an appropriate mass of H_2PtCl_6 was dissolved in DI water to achieve the desired weight loading. This Pt-containing solution was added dropwise to the CeO_2 to form a slurry. The slurry was mixed continuously in a mortar and pestle for 2 hours until the complete evaporation of the DI water was achieved. The powder was dried overnight in air at 60 °C and then reduced in a flowing stream of 5% H_2/Ar for 2 hours at 400 °C, yielding a black powdered sample of Pt/CeO_2. Bulk structural characterization was performed using X-ray diffraction (XRD) on a Bruker D-5000 with a Cu Kα source (λ = 0.15406 nm). The powder XRD patterns of the bare CeO_2 and Pt-loaded CeO_2 nanocubes are shown in **Supplementary Figure 1**. A simulated XRD pattern of an infinite crystal of CeO_2 is also shown for reference (*JCPDS* No. 34-0394). The peaks present in the XRD pattern for the bare CeO_2 nanocubes (blue line) match well with that of the simulated crystal (black line), indicating that the sample is phase-pure CeO_2 (space group Fm-3m, a = 5.41 Å). The XRD pattern for the Pt/CeO_2 nanocubes (red line) is essentially identical to that of the bare CeO_2 support.

The as-reduced Pt/CeO_2 catalyst powder was imaged in a probe-corrected JOEL ARM 200F scanning transmission electron microscope (STEM) operated at 200 kV. A TEM specimen was prepared by dry-loading the freshly reduced catalyst powder onto a holey carbon film. High angle annular dark field (HAADF) or so-called Z-contrast images were collected of many different areas of the TEM specimen and the Pt nanoparticle size distribution was determined by measuring the size of about 500 different Pt nanoparticles. **Supplementary Figure 2** displays two representative HAADF STEM images of the Pt/CeO_2 powder showing the dispersion of the Pt on the CeO_2 support along with a histogram of the size distribution measurement. The histogram of the size distribution measurement shows that the Pt nanoparticle population has an average nanoparticle size of 1.6 nm.

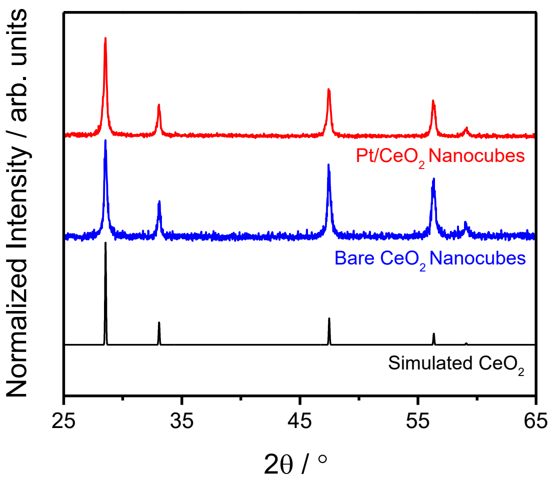

Supplementary Figure 1. Powder XRD patterns from (blue) bare CeO_2 and (red) Pt-loaded CeO_2 compared with (black) a simulated pattern from a perfect CeO_2 crystal.

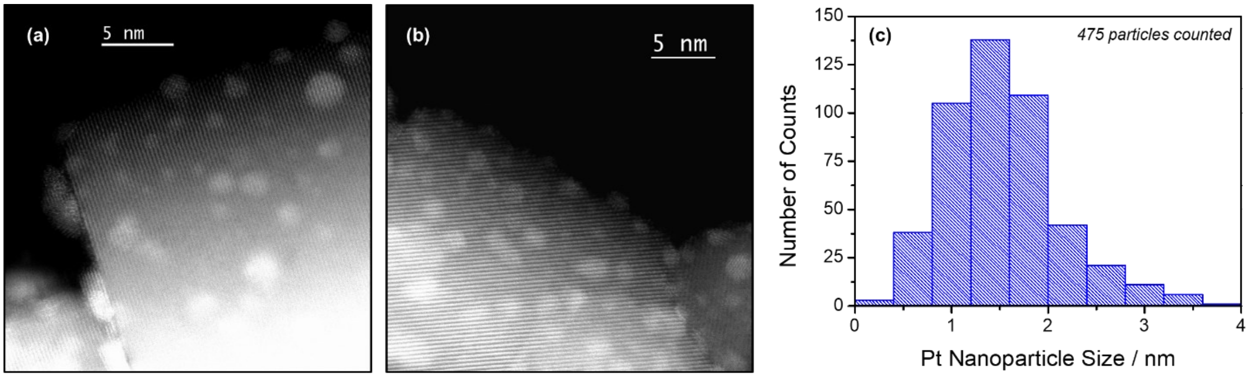

Supplementary Figure 2. (a, b) Z-contrast STEM images of a typical Pt-loaded CeO_2 nanoparticle imaged during the Pt nanoparticle size measurement, and **(c)** Pt nanoparticle size distribution histogram (n = 475) for the 17 wt.% Pt/CeO_2 catalyst, which exhibits an average Pt particle size of 1.6 nm.

The catalyst's activity for CO oxidation was evaluated in a packed bed plug flow reactor. Plug flow reactor experiments were performed in a RIG-150 micro-reactor from In Situ Research Instruments (ISRI). Effluent gas compositions were measured with a Varian 3900 gas chromatograph (GC) equipped with a thermal conductivity detector (TCD). In a typical reaction, 20 mg of catalyst powder was diluted on 2.6 g of inert SiO_2 sand particles having size of 1 mm and loaded into the reactor. A reducing treatment was preformed prior to the introduction of reactants, wherein 40 standard cubic centimeters per minute (SCCM) of 5% H_2/Ar was flown while the reactor bed was held at 400 °C for 2 hours. The reactor was then cooled to room temperature with continued 5% H_2/Ar flow. Upon cooling, the gas stream was switched to 8 SCCM of 10% CO/He and 22 SCCM of 5% O_2/He, balanced with 150 SCCM of pure He, which corresponded to a gas hourly space velocity of 12,500 hr^{-1}.

The reactor was then heated up to 400 °C at a rate of 1.5 °C/minute while the effluent gas composition was analyzed. He gas was used as a carrier gas. The reaction temperature was monitored by a thermocouple placed in the center of the catalyst bed. The CO conversion, X_{CO}, was quantified by calculating $X_{CO} = (CO_{in}-CO_{out})/CO_{in}$, where CO_{in} and CO_{out} denote the molar flowrate of CO into and out of the reactor, respectively. A reaction rate was calculated by multiplying the conversion by the molar flow rate of CO into the reactor. Turnover frequencies were computed by normalizing the reaction rates to the estimated number of Pt atoms at the metal-support interfacial perimeter, as determined by the derivation described in the next section of the Supplementary Information. We normalize to the number of Pt atoms at the perimeter as we assume that the reaction occurs through a Mars-van Krevelen mechanism at the perimeter of the metal-support interface. We note that this normalization assumes that each perimeter Pt atom is active and that the number of perimeter Pt atoms is identical both in vacuum conditions, where the number is counted, and under reaction conditions, where the catalysis takes place. Essentially, though, the TOF reported here is linearly related to the mass-normalized rate of product formation. Activation energies for CO oxidation were calculated through an Arrhenius analysis of the activity data taken in the low-conversion regime (i.e., < 25%). Figure 1a of the main text shows the light-off curves for CO oxidation. **Supplementary Figure 3** below shows the Arrhenius analysis of the rate data, which reveals that the Pt/CeO_2 catalyst shows an apparent activation energy E_a of 74 kJ/mol.

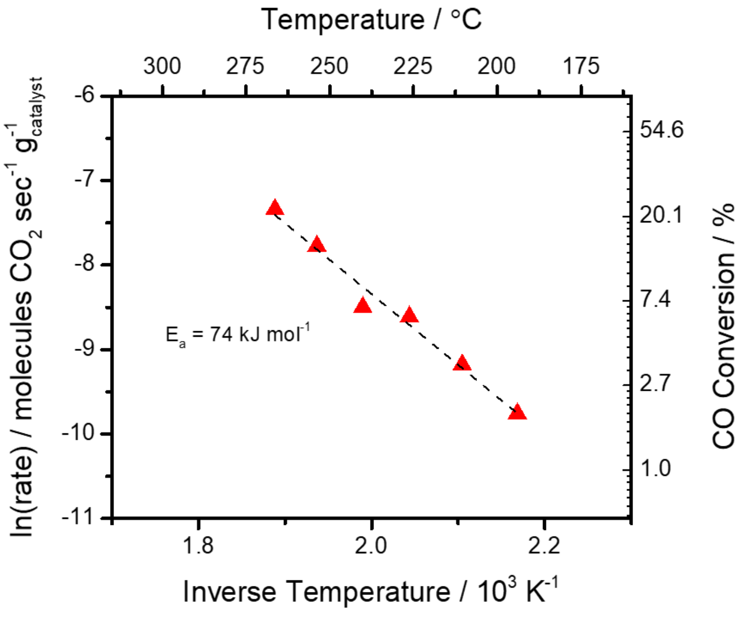

Supplementary Figure 3. Arrhenius analysis of the Pt/CeO_2 catalyst light-off conversion data (see Figure 1a of the main text) shows that the apparent E_a for CO oxidation is 74 kJ mol^{-1}.

Supplementary Note 2: Derivation of turnover frequency (TOF) on an interfacial perimeter-site basis from Pt nanoparticle size distribution

The number of atoms of Pt at the metal-support interfacial perimeter can be determined from the particle size histogram by assuming that the Pt nanoparticles have a hemispherical shape[2]. A derivation of this quantity and a sample calculation of the TOF for a given conversion are provided below. First, we define the TOF as:

(1) $$TOF\left[\frac{molecules\ CO_2}{perimeter\ site*second}\right] = \frac{F_{CO,in} \times X_{CO}}{m_{Pt/CeO_2} \times \gamma}$$

Where $F_{CO,in}$ represents the molar flowrate of CO into the *ex situ* reactor or the environmental cell, X_{CO} represents the CO conversion, $m_{Pt/CeO2}$ represents the mass of Pt/CeO_2 catalyst loaded into the *ex situ* reactor or *operando* pellet, and γ represents the number of atoms of Pt at the perimeter of the metal-support interface *per gram of Pt/CeO₂ catalyst*. The quantities $F_{CO,in}$ and $m_{Pt/CeO2}$ are controllable, and X_{CO} is determined experimentally. To calculate the TOF for a given X_{CO}, one simply requires an estimate of the number of perimeter sites per gram of catalyst, i.e., γ.

The value of γ was determined from HAADF-STEM measurements by assuming that the Pt nanoparticles have a hemispherical shape[2]. First, the volume, V_i, and interfacial perimeter length (i.e., circumference), L_i, was calculated for each particle *i* measured with diameter d_i, as described in the following equations:

(2) $$V_i = \frac{2}{3}\pi\left(\frac{d_i}{2}\right)^3$$ (3) $$L_i = \pi d_i$$

The total volume, V_{total}, and interfacial perimeter length, L_{total}, of all the particles measured in the data set is then computed as the sum of all *n* measurements (here $n = 475$):

(4) $$V_{total} = \sum_{i=1}^{n} V_i$$ (5) $$L_{total} = \sum_{i=1}^{n} L_i$$

Using the density of Pt, $\rho_{Pt} = 21.45$ g/cm^3, the total mass of Pt observed in the measurements is:

(6) $$m_{total} = V_{total} \times \rho_{Pt}$$

This allows for a mass-specific interfacial perimeter length, L'_{total}, to be calculated as:

(7) $$L'_{total} = L_{total} \div m_{total} \times 0.17\left[\frac{g_{Pt}}{g_{Pt/CeO_2}}\right]$$

The factor of 0.17 is included since the catalyst is 17% Pt by weight. The last quantity needed to calculate the number of perimeter Pt atoms per gram catalyst is a value for the width of a Pt atom, $d_{Pt\,atom}$. Given the mass density of Pt, ρ_{Pt}, we can estimate a value by first calculating the number of atoms in a cubic nm of Pt, *i.e.*, the atomic volume density, φ_{Pt}:

(8) $$\varphi_{Pt} = \rho_{Pt} \div M_{Pt} \times N_{Avogadro}$$

Where M_{Pt} is the molar mass of Pt, 195.08 g mol^{-1}, and $N_{Avogadro}$ is Avogadro's number, 6.02 × 10^{23} atoms mol^{-1}. The width of a Pt atom can be computed by inverting and taking the cube root of the atomic volume density:

$$(9) \qquad d_{Pt\,atom} = \left(\frac{1}{\varphi_{Pt}}\right)^{\frac{1}{3}}$$

And so, the number of perimeter Pt atoms per gram catalyst, γ, is:

$$(10) \qquad \gamma \; = L'_{total} \div d_{Pt\,atom}$$

Following this arithmetic and performing the analysis on the Pt nanoparticle size distribution plotted in **Supplementary Figure 2c**, a value for γ of 5.45 × 10^{19} sites g^{-1} is obtained. A TOF can now be calculated for a given conversion assuming the CO flowrate and mass of catalyst in the reactor are known. During the *operando* TEM experiment, around 180 ug of Pt/CeO_2 catalyst was loaded into the operando pellet. The inlet flow rate of CO into the cell was 0.08 SCCM or 5.94 × 10^{-8} mol CO sec^{-1}, which is 3.58 × 10^{16} molecules CO sec^{-1}. It should be briefly mentioned here that the calculated TOF will still need to be corrected by finite element simulations in order to accurately represent the reaction rate of catalyst particles supported on the TEM grid, which is discussed at length in the following section of the SI. Regardless of this additional complexity, for an example CO conversion of 10%, the *in situ* TOF would be calculated as:

$$(11) \qquad TOF = \frac{F_{CO,in} \times X_{CO}}{m_{Pt/CeO2} \times \gamma} = \frac{3.58 * 10^{16} \left[molecules\ CO\ sec^{-1}\right] \times 0.10}{180 * 10^{-6}\ [g] \times 5.45 \times 10^{19} \left[\frac{sites}{g}\right]} \approx 0.37 \left[\frac{molecules\ CO_2}{perimeter\ site * second}\right]$$

The TOFs reported in this document have all been calculated following this procedure.

Supplementary Note 3: Details of finite element simulation of *operando* ETEM reactor

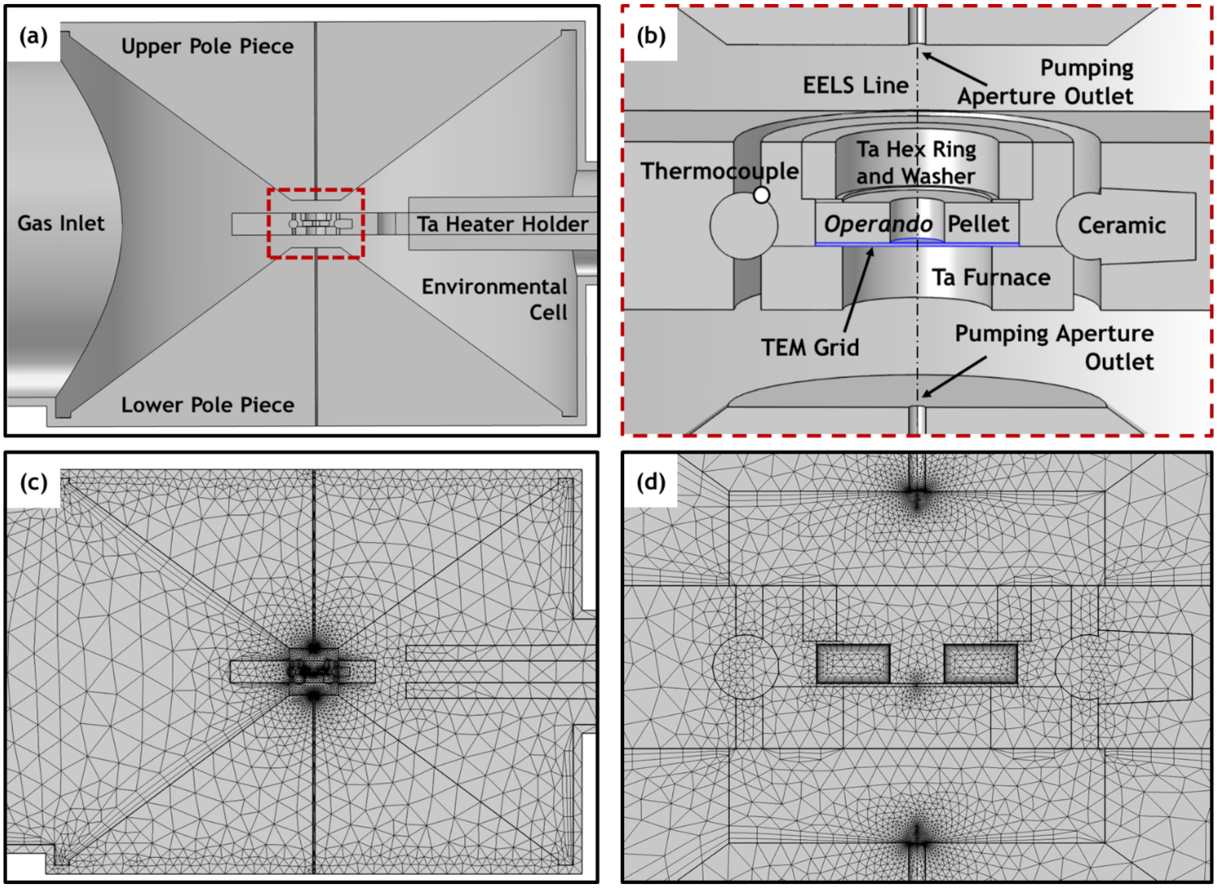

Supplementary Figure 4. Finite element model geometry and mesh in full view of the environmental cell **(a, c)** as well as in an enhanced view focused on the *operando* ETEM reactor **(b, d)**. In **(b)**, the TEM grid domain is colored blue for clarity. A defined pressure and composition of reactant gas flows into the cell from the inlet on the left. The differential pumping aperture outlets in the pole pieces serve as outlets. **(b)** The pellet reactor and furnace holder reside between the two pole pieces. A thermocouple on the outer edge of the Ta furnace is used to control the temperature of the reactor.

Finite element simulations of the *operando* ETEM reactor were performed in COMSOL Multiphysics® software using an adapted model developed previously by Vincent et al.[3]. The Computational Fluid Dynamics, Heat Transfer, and Chemical Reaction Engineering modules were used. The model geometry is shown in the top of **Supplementary Figure 4**, which displays (a) the environmental cell and (b) the *operando* ETEM pellet reactor. The TEM grid is highlighted in blue. The finite element mesh for the model is displayed in the bottom half of **Supplementary Figure 4**, which shows the mesh, for (c) the full cell and (d) the reactor. The mesh is made of around 205,000 elements total.

Steady-state simulations were performed under conditions nominally identical to the *operando* ETEM experiment described in the main text. A reactant gas inflow of CO and O_2 in a 1:1.375 ratio was admitted to the cell. The total inlet flowrate was adjusted to 0.08 standard cubic centimeters per minute (SCCM) in order achieve a cell static pressure of 0.57 Torr. Heat transfer and multicomponent mass transport were implemented as described in[3]. The reaction was modeled as 0th order, with an activation energy, E_a, of 74 kJ mol^{-1}, which was taken from the Arrhenius analysis done on the plug flow reactor data as presented in **Supplementary Figure 3**. The spatial distribution of catalyst in the pellet was modeled with an egg-shell profile. The furnace temperature was set in the range of 144 – 297 °C.

In the model, any quantity of interest (e.g., gas composition, temperature, reaction rate, etc.) may be determined at any element or averaged/integrated over chosen domains. For example, the rate of product formation (i.e., the reaction rate, with units of mol CO_2 per second) may be found by integrating the reaction rate throughout the domain of the *operando* pellet where the reaction occurs. Moreover, by integrating the rate along the inner surface of the pellet, where the composition and temperature are both nearly identical to that at the TEM grid, one can determine the reaction rate of the catalytic particles on the TEM grid[3]. As another example, the gas composition measured experimentally can be replicated as an integral of the composition along the path labeled “EELS Line” which represents the fast electron beam trajectory in the ETEM (see **Supplementary Figure 4b**). A line integral along the EELS line can be used to simulate the reactant conversion that one would measure experimentally with EELS. Here, we have employed the model to establish a framework that allows us to link the reactant conversion measured along the EELS line to the reaction rate of the catalyst that is imaged on the TEM grid.

In the model, the true rate of product formation may be found by integrating the reaction rate throughout the domain of the pellet. The rate may be normalized to mass by also integrating the mass distribution within the same domain. Here we set the total integrated mass of the catalyst in the pellet to be 180 µg, as this corresponds to the amount loaded experimentally. As mentioned, one can determine the mass-normalized rate for the catalytic nanoparticles on the TEM grid by integrating the mass and rate along the innermost surface of the pellet[3]. Here we integrate the mass and rate around a 50 µm thick layer at the surface of the inner hole in the pellet. This rate, which refer to as r_{grid}, is tabulated as function of temperature in **Supplementary Table 1**.

Experimentally, the rate of product formation may be estimated from the EELS CO conversion measurement. The estimated rate of CO_2 formation, r_{EELS}, may be calculated by multiplying the measured EELS CO conversion with the inlet molar flow rate of CO into the cell:

$$(12) \quad r_{EELS} = \frac{X_{CO} \times \dot{n}_{CO,in}}{m_{cat}}$$

Here, r_{EELS} is the estimated rate of CO_2 formation, X_{CO} is the CO conversion measurement derived from EELS, $\dot{n}_{CO,in}$ is the inlet molar flow rate of CO into the cell, and m_{cat} is the mass of catalyst loaded in the reactor. The mass-normalized rate of CO_2 formation estimated by the EELS CO

conversion measurement may also be simulated in the model, since it is possible to replicate the conversion measurement by integrating the composition along the EELS line. The mass-normalized rate estimated by the EELS conversion measurement in the model is tabulated as a function of temperature in **Supplementary Table 1**. The CO conversion values estimated through the EELS composition measurements are also given for reference.

It is of interest to compare the estimated – and experimentally measurable – rate of product formation, r_{EELS}, to the value which represents the rate at the TEM grid, r_{grid}. The last column of **Supplementary Table 1** presents the calculated ratio of the two values. Observe that the ratio of r_{EELS} to r_{grid} is not constant with conversion but varies by nearly 20% from 0.80 to 0.66 over the conditions explored here. Previously we have shown that under higher conversion conditions (e.g., X_{CO} > 0.70), the difference between the r_{EELS} and r_{grid} can grow beyond 200%, clearly demonstrating the need for and power of a model that allows one to relate the reactant conversion to the true reaction rate (i.e., activity) of the imaged catalyst. In the context of the present work, the true rate can be calculated correctly simply by scaling the rate measured through EELS by a conversion-dependent factor that is given in in the last column of **Supplementary Table 1**.

Supplementary Table 1. Summary of reaction rate analysis. The CO conversion estimated through the EELS measurement is presented as a function of the furnace temperature set point. The mass-normalized rate of CO_2 formation obtained by integrating the reaction rate and mass within a 50 µm thick layer at the surface along the inner hole in the pellet, r_{grid}, which represents the rate at the TEM grid, is presented, along with the rate estimated from the EELS CO conversion measurement, r_{EELS}. Finally, the ratio of the rate estimated through EELS and the rate at the TEM grid is given.

**Furnace Set Point (°C)**	X_{CO} **Estimated from EELS (%)**	r_{grid} **(µmol CO_2 sec^{-1} g_{cat}^{-1})**	r_{EELS} **(µmol CO_2 sec^{-1} g_{cat}^{-1})**	**Ratio of** $r_{EELS} : r_{grid}$ **(%)**
144	0.11	0.26	0.21	80%
202	1.26	3.48	2.44	70%
251	7.07	19.83	13.66	69%
261	9.65	27.20	18.65	69%
271	13.01	36.89	25.16	68%
275	14.61	41.55	28.25	68%
285	19.33	55.51	37.40	67%

297	26.61	77.57	51.53	66%

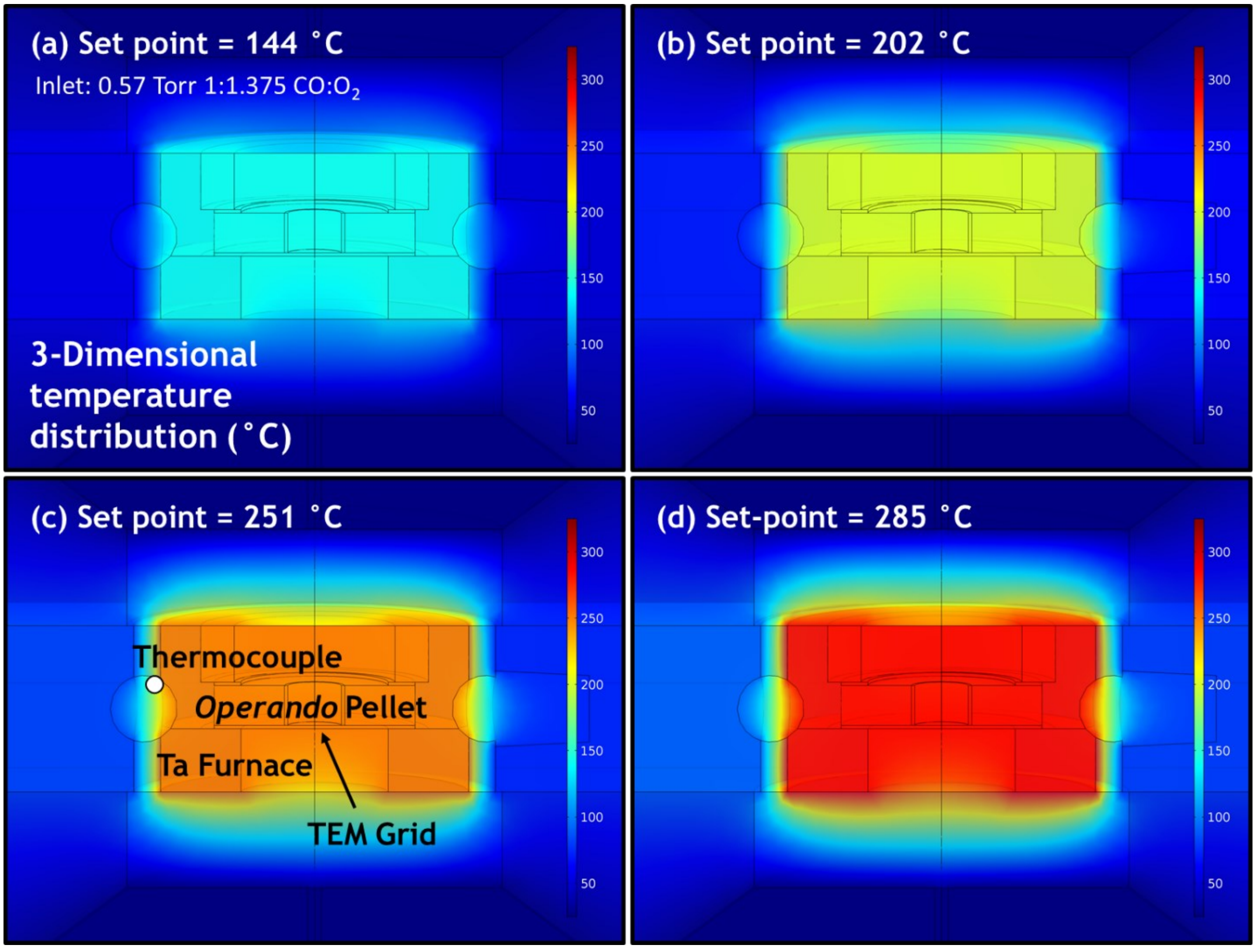

Supplementary Figure 5. Temperature distribution in and around the ETEM reactor during catalysis for furnace thermocouple set points of **(a)** 144 °C, **(b)** 202 °C, **(c)** 251 °C, and **(d)** 285 °C. At each condition the temperature distribution is largely uniform and matches well with the set point.

In addition to establishing a relationship between the measured conversion and the quantitative chemical kinetics of the catalyst, it is also important to investigate the extent of any thermal gradients that may exist within the reactor. **Supplementary Figure 5** displays the 3-dimensional temperature distribution in and around the *operando* ETEM reactor for four furnace thermocouple set points: of (a) 144 °C, (b) 202 °C, (c) 251 °C, and (d) 285 °C. Note that these correspond to conditions where the catalyst is active and producing CO_2. These temperatures were chosen to investigate as they correspond to those explored during the *operando* experiment.

Observe that the temperature distributions appear largely uniform in the hot zone of the reactor where the catalyst is located. These results are qualitatively similar to those reported previously[3,4], and they are expected given the large thermal mass of the furnace holder. A quantitative comparison of the temperature difference between the furnace thermocouple set point and the average temperature at the TEM grid is presented below in **Supplementary Table 2**. For the conditions explored here, the average temperature on the TEM grid surface differs from the set point by < 2 °C. The temperature at the grid is slightly less, as expected. Overall, these results indicate that the *operando* ETEM reactor can be treated as essentially isothermal and they suggest that the furnace thermocouple can be used as a reliable probe of the temperature surrounding the imaged catalyst.

Supplementary Table 2. Quantitative investigation of the temperature distribution in the *operando* ETEM reactor. The furnace thermocouple set point is compared to the actual temperature at the TEM grid, which contains the catalyst that is imaged during an experiment. The maximum and minimum temperatures are given along with the average temperatures evaluated at the inner surface that coincides with the electron beam optic axis within the *operando* pellet.

Furnace Set Point (°C)	Max Temperature at Inner Grid Surface (°C)	Min Temperature at Inner Grid Surface (°C)	Average Temperature at Inner Grid Surface (°C)	Difference between Set Point and Average Grid Temperature (°C)
144	143.43	143.34	143.38	0.62
202	201.10	200.97	201.02	0.98
251	249.80	249.63	249.69	1.31
261	259.74	259.56	259.63	1.37
271	269.68	269.49	269.56	1.44
275	273.65	273.46	273.53	1.47
285	283.59	283.39	283.47	1.53
297	295.53	295.32	295.40	1.60

Supplementary Note 4: Time-averaged image methodology

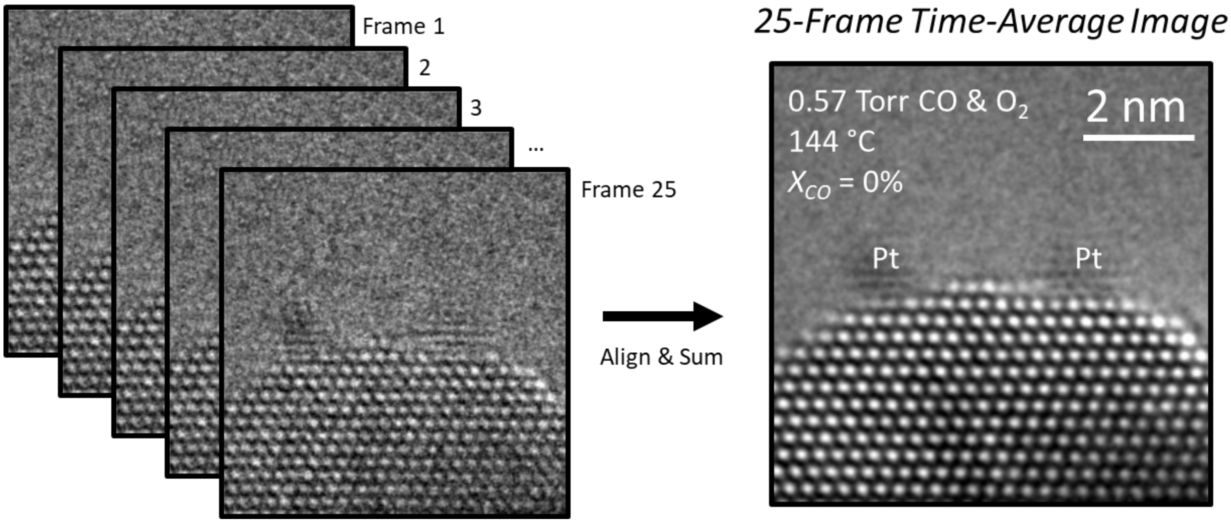

Supplementary Figure 6. Schematic of time-averaged image methodology. The limited electron flux of about 10^3 e^- Å^{-2} s^{-1} used here results in a poor SNR in each individual 0.5 s exposure frame (left). To increase the SNR in the image without increasing the electron dose, multiple independent frames were selected, aligned, and summed together to produce a time-averaged image (right). Individual images were selected on the basis of bulk Ce column visibility to avoid artifacts from drift in the sample or electron optics. The time-average shown at right was constructed from 25 frames, which yields a 12.5 s time-average. The time-averaged image exhibits a marked increase in SNR available for local structural analysis.

Supplementary Note 5: Multislice TEM image simulations of contrast reversals in CeO_2-supported Pt nanoparticles undergoing rigid-body rotations

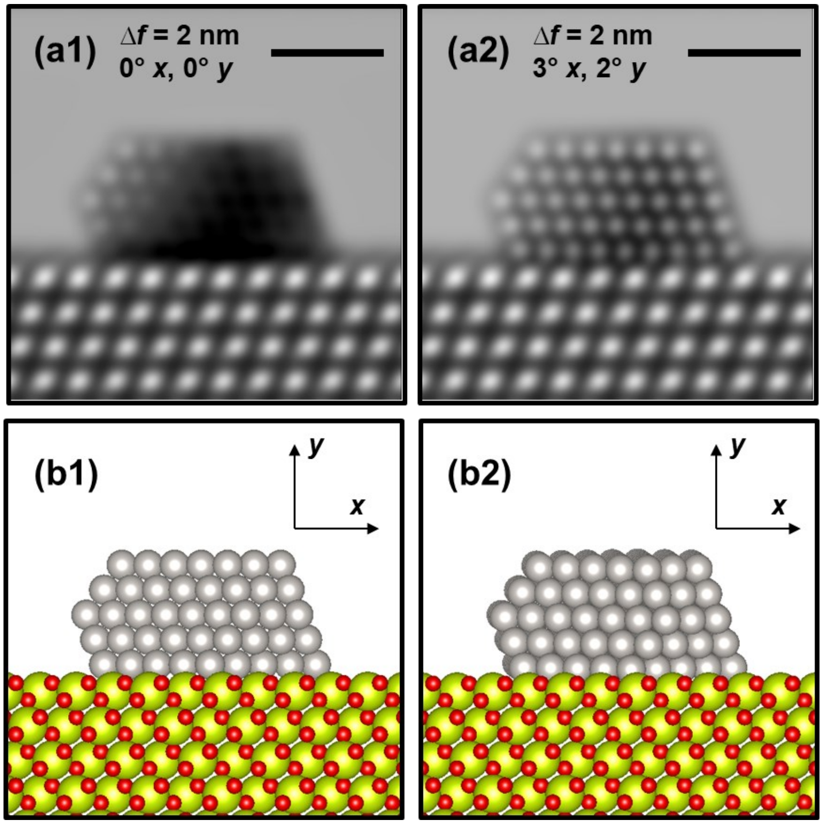

Supplementary Figure 7. Multislice TEM image simulations of a Pt nanoparticle supported on a CeO_2 (111) surface demonstrating the appearance of mixed black and white Pt atomic column contrast and contrast reversals due to rigid body rotations. Figure **(a1)** presents a TEM image simulation of a Pt particle that is oriented along the incident electron beam without any tilt, calculated for an electron optical defocus of 2 nm. Notice the appearance of mixed black and white atomic column contrast in the Pt nanoparticle due to a contrast reversal occurring between the thinner sites on the edge of the particle and the thicker sites in the particle center. Figure **(a2)** presents another simulation at the same defocus but now for a Pt particle that has been tilted by three degrees about the horizontal *x* axis and by two degrees about the vertical *y* axis (see **(b1)** and **(b2)** for the atomic models as well as the orientation of the axes). In the case that the particle is tilted, the image simulated at a defocus of 2 nm no longer shows a mixture of black and white atomic column contrast but instead shows only white atomic column contrast, presumably due to a disruption in the electron beam channeling conditions. Simulations performed at other comparable tilts (i.e., of a few degrees) show similar behavior. Simulations were performed in the Dr. Probe software package[5], using an accelerating voltage of 300 kV, a beam convergence angle of 0.2 mrad, a focal spread of 4 nm, a 3rd order spherical aberration of -13 µm, a 5th order spherical aberration coefficient of 5 mm, and a defocus of 2 nm. During the simulation, the CeO_2 support was tilted independently by 1.5° *x* and 1.0° in *y*. The tilt information in the figure inset of **(a1)** and **(a2)** therefore denote the orientation of the Pt with respect to the incident electron beam. Scale bars in **(a1)** and **(a2)** correspond to 2 nm. An isotropic vibration envelope of 85 pm was applied during the image calculation. In the models, Pt is gray, Ce is yellow-green, and O is red.

1. Analysis of Bragg spots appearing in Fourier transform of Pt nanoparticles imaged under CO oxidation reaction conditions

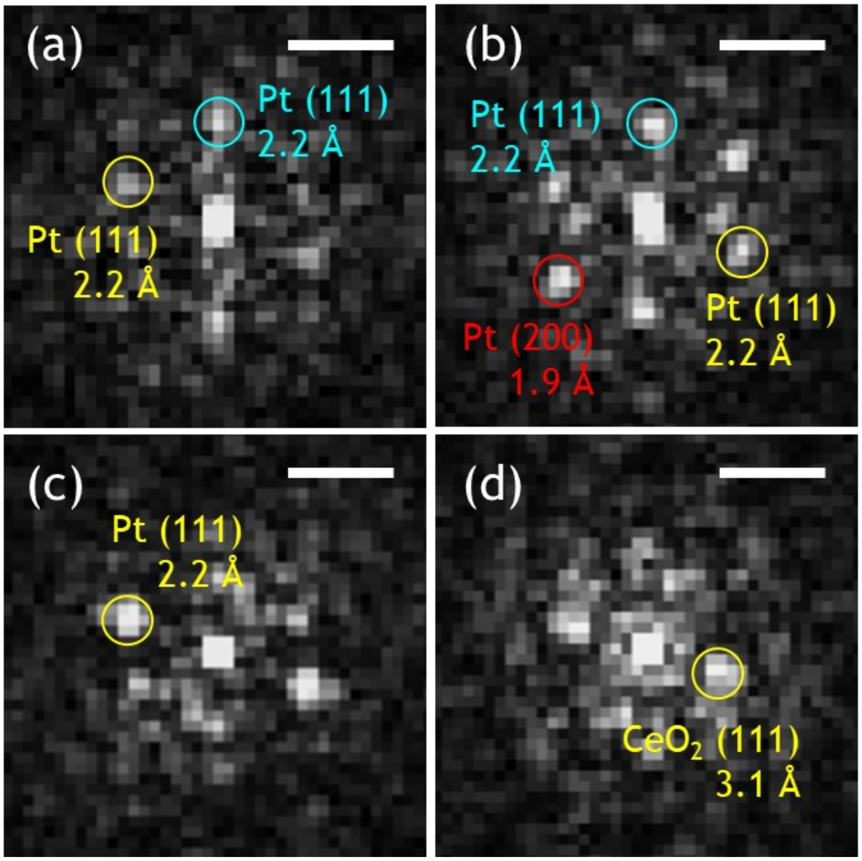

Supplementary Figure 8. Indexing major Fourier transform (FT) spots shown in Figure 2(f1) - (f4) in the main text. Here, **(a)** corresponds to (f1), **(b)** to (f2), and so on. Major FT spots corresponding to the (111) and/or (200) lattice plane spacings of the Pt metal phase can be seen in **(a)**, **(b)**, and **(c)**, while in a spot corresponding to the CeO_2 (111) lattice plane spacing is discernable in **(d)**. Color coding is simply for clarity. The scale bar in each subfigure corresponds to 5.0 nm^{-1}.

Supplementary Note 6: Dynamic structural response of CeO_2-supported Pt nanoparticles at elevated temperatures

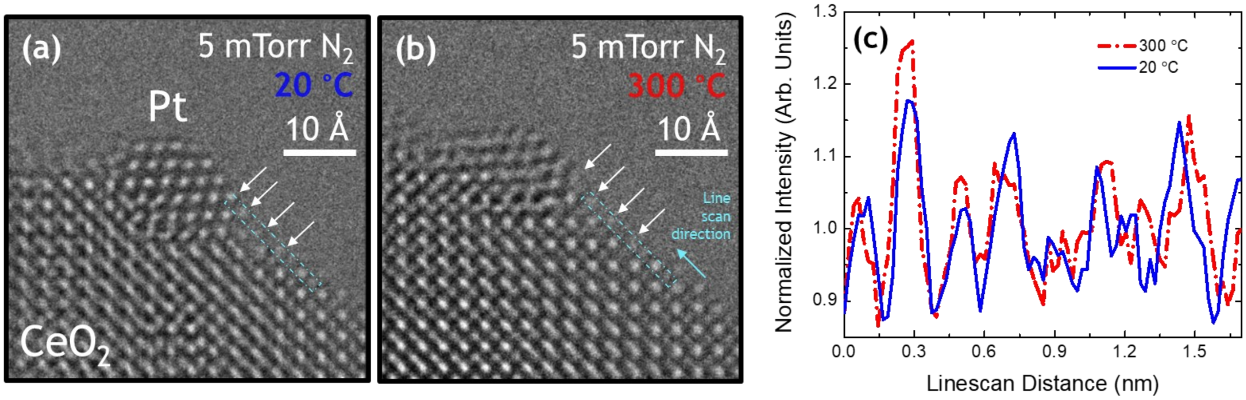

Supplementary Figure 9. Half-second exposure *in situ* ETEM images of the same nanoparticle of a Pt/CeO_2 catalyst in 5 mTorr N_2 at **(a)** 20 °C and **(b)** 300 °C. Clearly resolved atomic columns bridging the Pt/CeO_2 three-phase boundary (TPB) are indicated with white arrows. In **(c)** a 1.2 Å wide integrated intensity line profile taken across the TPB from the region and along the direction indicated in the images shows that the atomic column contrast at 20 °C differs little from that observed when the catalyst is heated up to 300 °C. These results suggest that both the loss of Pt fringe visibility and the evolution of Ce column blurring observed during catalysis are not attributed strictly to thermal effects, but instead to dynamic chemical/structural processes resulting from catalytic turnover.

The analysis of the time-averaged *operando* TEM images shows that the turnover frequency for CO oxidation is correlated with structural dynamics taking place in the Pt nanoparticles and at/near the three-phase boundary (TPB). However, as the catalyst must be heated in order to be activated, it may be reasoned that the dynamics are driven by the elevated temperature of the catalyst and not the catalytic chemistry. We have therefore performed *in situ* TEM imaging experiments in inert gases at elevated temperatures, in order to investigate what may be called spectator fluxional behavior attributed only to the presence of applied heat and not to catalytic surface chemistry. **Supplementary Figure 9** presents 0.5 s exposure *in situ* ETEM images of the same CeO_2-supported Pt nanoparticle in 5 mTorr of inert N_2 gas at (a) 20 °C and (b) 300 °C. Note that 300 °C is nearly the highest temperature explored in the *operando* ETEM experiment (297 °C).

Compared to the observations made *in situ* under reaction conditions (e.g., Figure 2 of the main text) or the case in which the catalyst is exposed to reactant gases and actively producing CO_2 (see e.g., Figure 4 of the main text), the catalyst exhibits a significantly different and more attenuated dynamic structural response as the temperature is elevated from 20 °C to 300 °C in an atmosphere of inert N_2. For instance, the Pt nanoparticles were observed to undergo fluxional dynamics that resulted in a loss of Pt lattice fringe visibility. As can be seen in **Supplementary Figure 9**, at room temperature the Pt nanoparticle is well-oriented into a [110] zone axis. Clear atomic-column

contrast is visible in the Pt as well as at the interfacial sites that bridge the right side of the Pt/CeO_2 perimeter (white arrows). The left side of the three-phase boundary yields contrast that is challenging to interpret due to thickness/tilt issues as well as the apparent overlap of the Pt and CeO_2 lattices. Nevertheless, the sharp visibility of the arrowed interfacial sites and Pt atomic columns indicates that the Pt nanoparticle, three-phase boundary, and nearby free CeO_2 surface exhibit general structural stability (fluxional dynamics, in comparison, would give rise to distinct motion artifacts, such as blurring or a localized loss of contrast). A 0.5 s exposure *in situ* ETEM image of the same Pt nanoparticle at an elevated temperature of 300 °C is shown in **Supplementary Figure 9b**. At elevated temperature, the Pt nanoparticle has now tilted into an oblique orientation near the [100] zone axis. Regardless of the static restructuring that may have occurred (presumably to lower the overall system energy at this higher temperature), the primary observation of importance for the present study is the lack of blurring and/or localized motion artifacts associated with fluxional dynamics. In this case, multiple Pt lattice fringes remain sharply resolved in the nanoparticle and atomic columns can even be seen on the Pt surface. Images acquired at different times during the observation also show well-resolved Pt lattice fringes and sharp atomic columns (see, e.g., **Supplementary Figure 10**), suggesting that the loss of Pt lattice fringe visibility observed under *operando* conditions is attributed to structural dynamics driven by catalytic surface chemistry. A close inspection of the images reveals that the particle occasionally undergoes rigid-body rotations of a few degrees (compare, e.g., **Supplementary Figure 9b** with **Supplementary Figure 10b**). Notably, such behavior was also seen to occur at room temperature (compare, e.g., **Supplementary Figure 9a** with **Supplementary Figure 10a**). This type of slow rotational behavior is completely different than the rapid dynamic structural fluctuations that we have observed during the experiments reported in the main text (see, e.g., Figure 2 or Figure 4) or during *in situ* TEM imaging experiments that we have performed of CeO_2-supported Pt nanoparticles exposed to CO[6], CO and O_2[7], and CO and H_2O[8].

A second dynamic structural behavior observed during the *operando* TEM experiment was the fluxionality of Ce atomic columns located at the three-phase boundary (TPB) and along the nearby free CeO_2 support surface. Here, we observe in inert N_2 that the atomic columns located at/near the three-phase boundary show similar contrast at 20 °C and 300 °C. For example, in **Figures 9a/b** and **Supplementary Figures 10a/b**, white arrows have been used to indicate a series of atomic columns that bridge the free CeO_2 support surface across the TPB to the Pt NP surface. Close inspection of the images and an examination of the intensity profile across this region reveal that the contrast and the width of the atomic columns are alike at low and elevated temperature. **Figure 9c** presents integrated intensity line profiles taken from a 1.2 Å wide window across the TPB from the region indicated by the dashed box in the corresponding images. The intensity line profiles have been normalized to the average vacuum intensity; the profile from 20 °C is plotted as a solid blue line, while the one from 300 °C is plotted as a dashed red line. A direct comparison of the magnitude and the shape of the profiles demonstrates that the contrast of the atomic columns at/near the Pt/CeO_2 TPB remains relatively unchanged as the catalyst is heated from 20 °C to 300 °C in N_2. These results suggest that the blurring of atomic sites at/near the Pt/CeO_2 TPB observed

during the operando TEM experiment arises from structural dynamics which do not occur in the absence of reactants.

Finally, we investigate the lattice plane separation distance of the top layer on the CeO_2 support near the Pt/CeO_2 interface. The surface lattice plane separation was measured from intensity line profiles taken from the bulk of the CeO_2 toward the surface; the region over which the profiles were taken at 20 °C and 300 °C are indicated in **Supplementary Figures 11a/b**, and the intensity profiles are themselves given in **Supplementary Figure 11c**. Measurements of the surface and the sub-surface lattice plane separation distance yield 1.98 ± 0.1 Å at both temperatures. Here we note that these measurements match those obtained from the bulk of the nanoparticle and that they agree with the accepted CeO_2 (110) Miller plane spacing of 1.91 Å. Thus, the outward CeO_2 surface relaxation observed under *operando* conditions also appears to be a consequence of structural dynamics that are driven by catalytic chemistry and not heating to 300 °C alone.

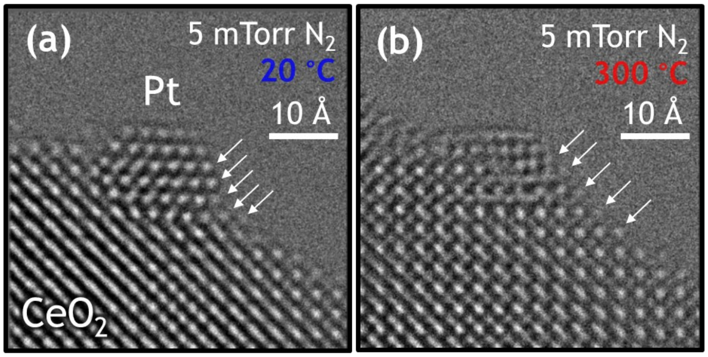

Supplementary Figure 10. Additional half-second exposure *in situ* ETEM images of the same nanoparticle of a Pt/CeO_2 catalyst in 5 mTorr N_2 at **(a)** 20 °C and **(b)** 300 °C. Clearly-resolved atomic columns bridging the Pt/CeO_2 TPB are indicated with white arrows.

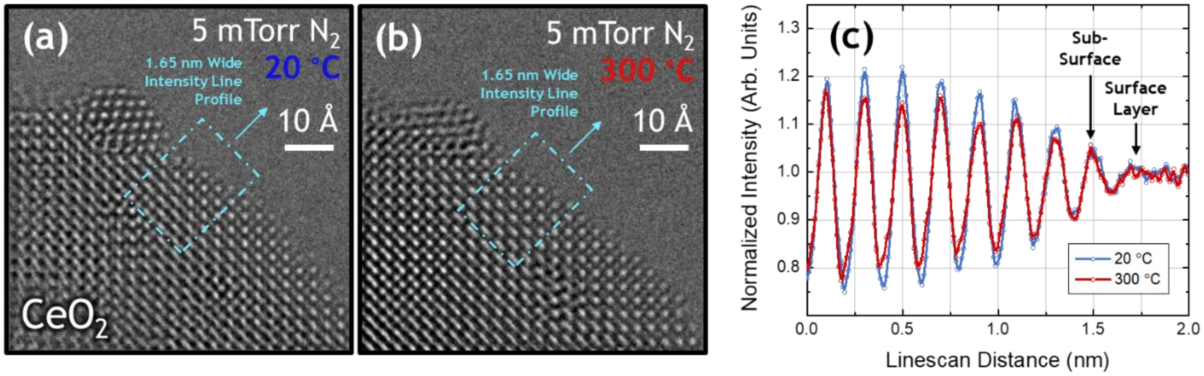

Supplementary Figure 11. Half-second exposure *in situ* ETEM images of the same nanoparticle of a Pt/CeO_2 catalyst in 5 mTorr N_2 at **(a)** 20 °C and **(b)** 300 °C, which were presented in Supplementary Figure 8 and which are shown here again to indicate the regions over which integrated intensity line profiles were taken to measure the surface lattice plane separation distance. The regions are indicated with light blue dashed boxes; the profiles were taken in the arrowed direction which lies perpendicular to the CeO_2 (110) surface. In **(c)** the intensity line

profiles at 20 °C (blue line) and 300 °C (red line) are plotted overtop one another. The line profiles were normalized to the average vacuum intensity; original data points are plotted as circles along with an interpolated spline function for clarity. The surface layer and the sub-surface lattice plane are indicated in the graph.

Supplementary Note 7: Additional *in situ* ETEM image time-series of CeO_2-supported Pt nanoparticles under CO oxidation reaction conditions

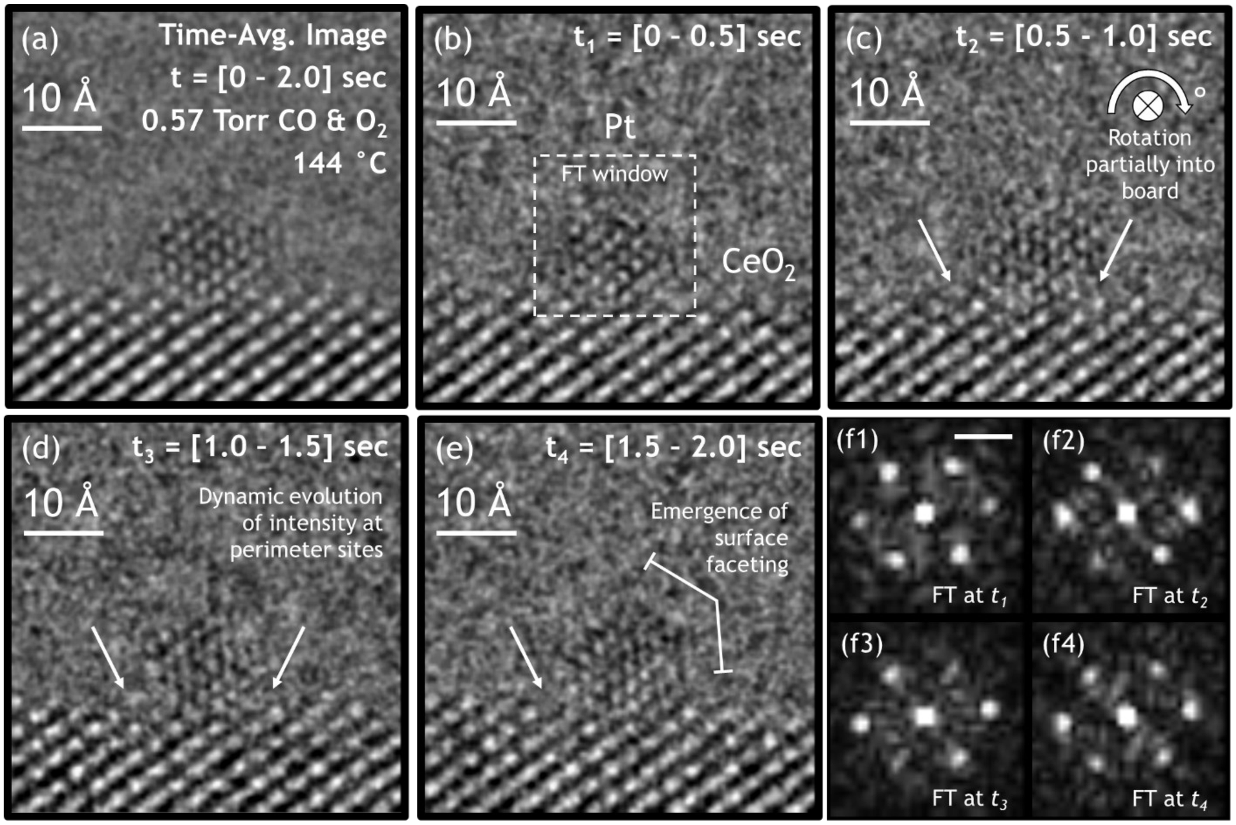

Supplementary Figure 12. *In situ* ETEM image time-series of CeO_2-supported Pt NP at 144 °C in 0.57 Torr of CO and O_2. Part **(a)** shows the time-averaged image of the catalyst, obtained by summing together the individual 0.5 second exposure frames over the entire over [0 – 2.0] second acquisition period. Parts **(b)** – **(e)** show the atomic-scale structural dynamics that evolve over 0.5 second intervals from $t = 0$ seconds to $t = 2.0$ seconds. Parts **(f1) – (f4)** display the FT taken at each time interval from the windowed region around the Pt NP, as denoted in (b). The scale bar in (f1) is 5.0 nm^{-1}. Images have been processed with a bandpass filter for clarity. FTs were produced from unfiltered, windowed images that were processed with a Hanning function to remove edge artifacts caused by windowing; the modulus of the FT is shown.

Supplementary Note 8: Analysis of intensity line profiles to quantify Ce atomic column blurring and CeO_2 (111) outward surface relaxation

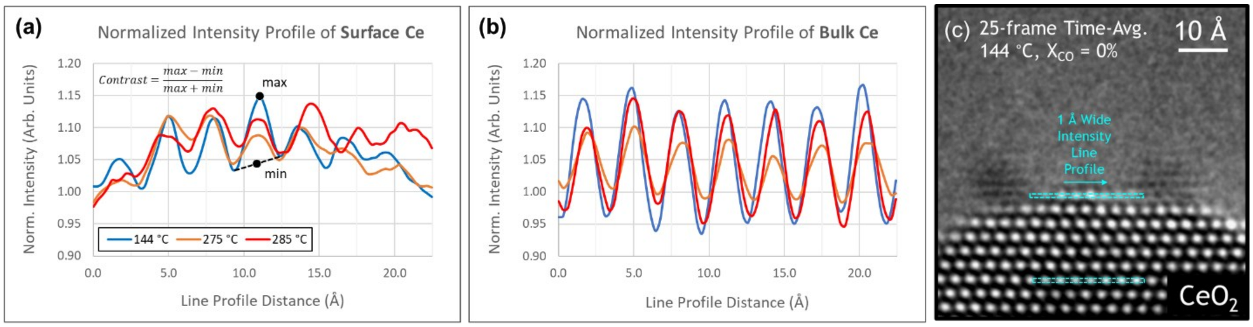

Supplementary Figure 13. Normalized intensity line profiles taken from **(a)** the free surface Ce columns between the Pt nanoparticles and from **(b)** a row of Ce cation columns several layers into the bulk of the nanoparticle. All of the intensity line profiles have been normalized to the average vacuum intensity at each condition. Intensity profiles are given for 144 °C (blue), 275 °C (orange), and 285 °C (red). The line profiles were generated over an integration window 92.1 pm wide (i.e., 3 pixels). The profile windows and direction are indicated in **(c)**. In (a) the equation for calculating the contrast of a column is given, and the process for determining the maximum and minimum values is demonstrated for the middle surface Ce column at 144 °C. In (b) the fact that the CeO_2 particle has tilted at 275 °C is evident by the reduction in contrast of the orange curve. When the catalyst was heated to 285 °C, the crystal tilted back, seen by the recovered contrast in the red curve.

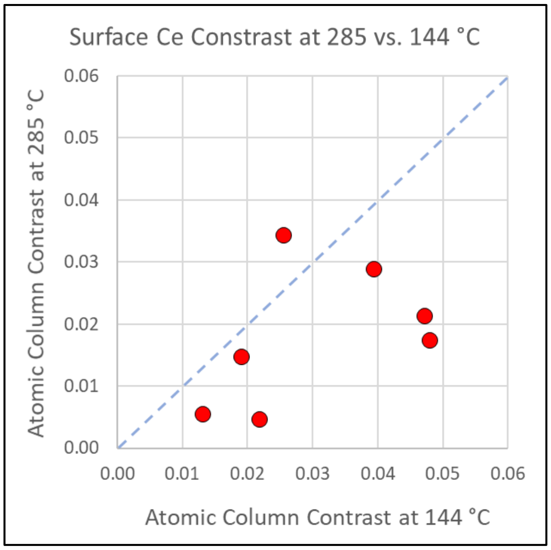

Supplementary Figure 14. The contrasts of the surface Ce columns at 24% CO conversion are plotted against the contrasts of the same column at 0% CO conversion. A straight dashed line is provided for reference, which shows that most Ce columns become more blurred with increasing conversion, as most points lie below the straight dashed line.

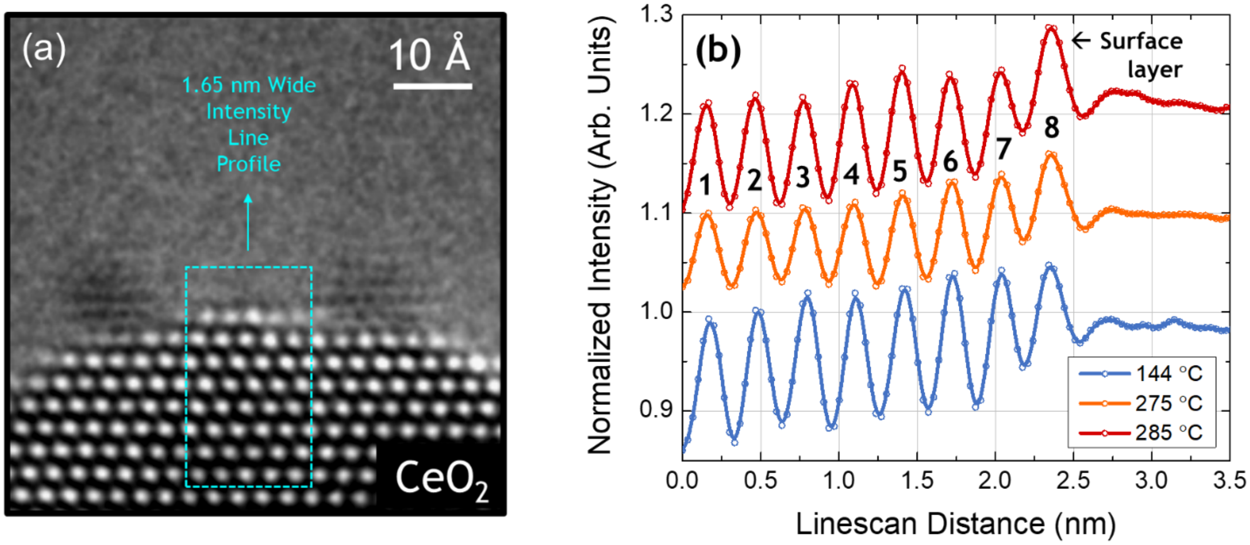

Supplementary Figure 15. Normalized intensity line profiles taken from interior of nanoparticle toward catalyst surface, revealing the outward CeO_2 (111) surface relaxation with increasing catalytic turnover. In **(a)** the region and direction of the intensity line scans are indicated for an example *operando* TEM time-averaged image acquired at 144 °C. The same region was used for all three conditions. In **(b)** the normalized intensity line profiles are plotted for 144 °C (blue), 275 °C (orange), and 285 °C (red). Original data points are plotted along with an interpolated spline which was used to measure the distances between the peaks in the profile. Numerical labels are given to the peaks to identify the measured separation distances that are provided in Supplementary Table 3.

Supplementary Table 3. Measurement of peak separation distances from intensity line profiles presented in Supplementary Figure 15. The peaks being measured are referenced with the numerical labels given in Supplementary Figure 15b (top). The outward relaxation of the CeO_2 (111) surface layer is presented in the last row of the table. The standard deviation of the measurements taken of all layers except the bulk (second to last row of the table) is used as an error bar on the measurement at each condition. Note that the averages of the sub-surface and bulk layer separation distances (third to last row of the table) are all close to the accepted CeO_2 (111) Miller plane spacing of 312 pm.

**Peaks Being Measured**	**Separation Distance at 144 °C (pm)**	**Separation Distance at 275 °C (pm)**	**Separation Distance at 285 °C (pm)**
1 – 2	305	313	307
2 – 3	313	307	306
3 – 4	308	313	310
4 – 5	311	309	315
5 – 6	303	314	307
6 – 7 (sub-surface)	312	312	319
Average of all layers except the surface	309	311	311
Std. dev. of all layers except the surface	4	3	5
**7 – 8 (surface layer)**	**306**	**315**	**320**

Supplementary References

1. Mai, H. X. *et al.* Shape-selective synthesis and oxygen storage behavior of ceria nanopolyhedra, nanorods, and nanocubes. *Journal of Physical Chemistry B* **109**, 24380–24385 (2005).
2. *Handbook of Heterogeneous Catalysis*. (Wiley-VCH Verlag GmbH). doi:10.1002/9783527619474.fmatter. (1097)
3. Vincent, J. L., Vance, J. W., Langdon, J. T., Miller, B. K. & Crozier, P. A. Chemical Kinetics for Operando Electron Microscopy of Catalysts: 3D Modeling of Gas and Temperature Distributions During Catalytic Reactions. *Ultramicroscopy* **218**, 113080 (2020).
4. Mølgaard Mortensen, P., Willum Hansen, T., Birkedal Wagner, J. & Degn Jensen, A. Modeling of temperature profiles in an environmental transmission electron microscope using computational fluid dynamics. *Ultramicroscopy* **152**, 1–9 (2015).
5. Barthel, J. Dr. Probe: A software for high-resolution STEM image simulation. *Ultramicroscopy* **193**, 1–11 (2018).
6. Crozier, P. A., Lawrence, E. L., Vincent, J. L. & Levin, B. D. A. Dynamic Restructuring during Processing: Approaches to Higher Temporal Resolution. *Microscopy and Microanalysis* **25**, 1464–1465 (2019).
7. Vincent, J. & Crozier, P. Atomic-resolution Operando and Time-resolved In Situ TEM Imaging of Oxygen Transfer Reactions Catalyzed by CeO_2-supported Pt Nanoparticles. *Microscopy and Microanalysis* **26**, 1694–1695 (2020).
8. Li, Y. *et al.* Dynamic structure of active sites in ceria-supported Pt catalysts for the water gas shift reaction. *Nature Communications* **12**, 914 (2021).
